# On the duration of the microbial lag phase

**DOI:** 10.1007/s00294-019-00938-2

**Published:** 2019-01-21

**Authors:** Lieselotte Vermeersch, Gemma Perez-Samper, Bram Cerulus, Abbas Jariani, Brigida Gallone, Karin Voordeckers, Jan Steensels, Kevin J. Verstrepen

**Affiliations:** 1 VIB Laboratory for Systems Biology, VIB-KU Leuven Center for Microbiology, Gaston Geenslaan 1, 3001 Leuven, Belgium; 2 CMPG Laboratory of Genetics and Genomics, Department M2S, KU Leuven, Gaston Geenslaan 1, 3001 Leuven, Belgium; 3 Department of Plant Biotechnology and Bioinformatics, Ghent University, Technologiepark 927, 9052 Ghent, Belgium; 4 VIB Center for Plant Systems Biology, Technologiepark 927, 9052 Ghent, Belgium

**Keywords:** Lag phase, *Saccharomyces cerevisiae*, Crabtree effect, Cellular memory, Gene regulation, Fermentation–respiration

## Abstract

When faced with environmental changes, microbes enter a lag phase during which cell growth is arrested, allowing cells to adapt to the new situation. The discovery of the lag phase started the field of gene regulation and led to the unraveling of underlying mechanisms. However, the factors determining the exact duration and dynamics of the lag phase remain largely elusive. Naively, one would expect that cells adapt as quickly as possible, so they can resume growth and compete with other organisms. However, recent studies show that the lag phase can last from several hours up to several days. Moreover, some cells within the same population take much longer than others, despite being genetically identical. In addition, the lag phase duration is also influenced by the past, with recent exposure to a given environment leading to a quicker adaptation when that environment returns. Genome-wide screens in *Saccharomyces cerevisiae* on carbon source shifts now suggest that the length of the lag phase, the heterogeneity in lag times of individual cells, and the history-dependent behavior are not determined by the time it takes to induce a few specific genes related to uptake and metabolism of a new carbon source. Instead, a major shift in general metabolism, and in particular a switch between fermentation and respiration, is the major bottleneck that determines lag duration. This suggests that there may be a fitness trade-off between complete adaptation of a cell’s metabolism to a given environment, and a short lag phase when the environment changes.

**Electronic supplementary material:**

The online version of this article (10.1007/s00294-019-00938-2) contains supplementary material, which is available to authorized users.

## The lag phase: a time for adapting gene regulation

The seminal work of François Jacob and Jacques Monod revealed how microbes temporarily stop dividing when they encounter a shift in nutrients (Jacob and Monod 1961). They hypothesized that this so-called lag phase allows cells to adapt to the new conditions by inducing the expression of genes needed for growth in the new environment. Their discovery was awarded the 1965 Nobel Prize in Physiology or Medicine and effectively started the field of gene regulation. Like this seminal work, the majority of subsequent studies on the lag phase have focused on carbon source switches, and while the details of the mechanisms underlying gene regulation and adaptation have since been largely uncovered, little attention has gone to investigating the speed at which these processes take place. In other words, we know surprisingly little about what determines the duration of the lag phase.

**Fig. 1:**
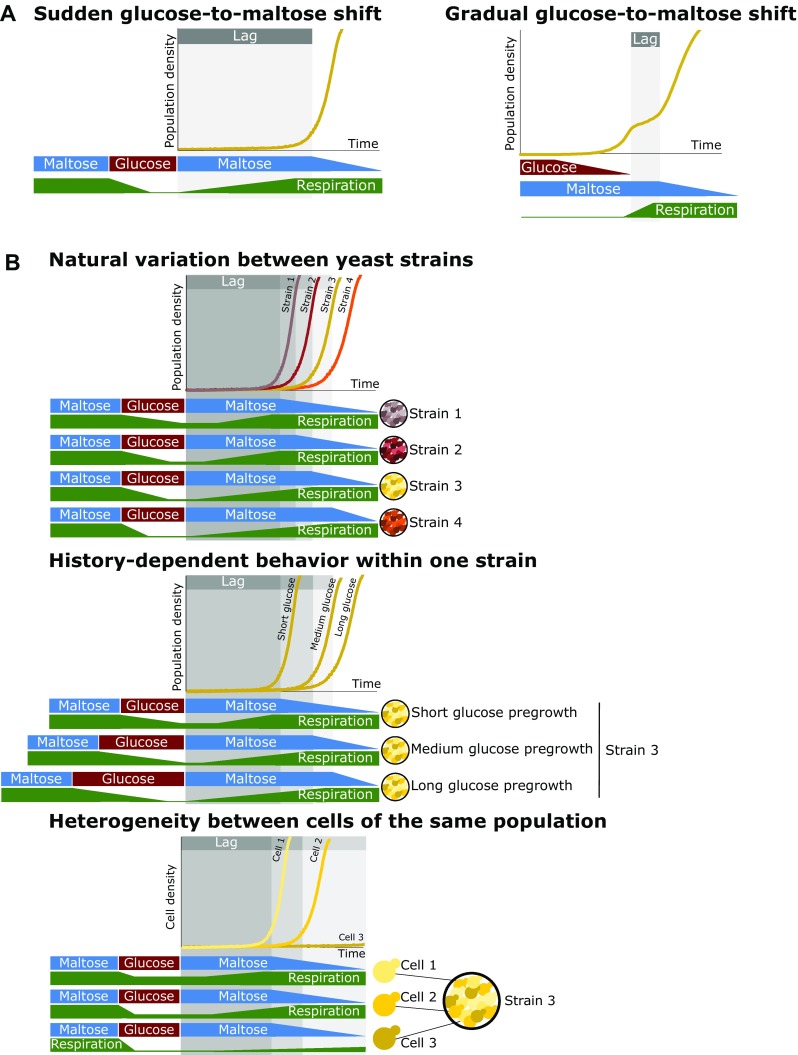
Overview of the different aspects of the lag phase and its link to respiration. **a** General experimental setups for measuring lag times. Left: cultures adapted to growth on maltose are transferred to glucose. After a specific time on glucose, cultures are washed into maltose and experience a lag phase. When transferred from maltose to glucose, the yeast cells induce glucose repression of the respiratory metabolism. Upon transfer to maltose, cells induce respiration to efficiently escape the lag phase to maltose. Right: cultures adapted to glucose are transferred to low-glucose media supplemented with maltose. Glucose is preferentially consumed, and upon depletion, cells experience a lag phase before growing on the available maltose. During glucose growth, respiratory metabolism is repressed. Upon depletion of glucose, respiration is induced to efficiently start growing on maltose. **b** Top: the natural variation in lag times between different *S. cerevisiae* strains correlates with the level of glucose repression of the respiratory metabolism. Middle: history-dependent behavior within one strain shows that the lag time depends on the time grown in glucose. Longer growth periods in glucose allow for more complete repression of respiration and thus give rise to longer lag phases upon a shift to maltose. Bottom: heterogeneity in lag times within an isogenic population. Cell density in figure is derived from colony size measurements. Within a population, some cells show stronger repression of respiration and thus longer lag phases, whereas other cells have more relaxed repression and thus show shorter lag phases

Two recent studies measured the lag phase when yeast cells are transferred from glucose to a less-preferred carbon source such as maltose, galactose or ethanol (Fig. 1a) (Perez-Samper et al. 2018; Cerulus et al. 2018). The results show large differences in lag duration between different yeast strains and even between individual cells of the same isogenic population.

What determines the length of the lag phase? The classic view is that the lag phase allows cells to adapt the expression of specific genes. In particular, it is often assumed that expression of transporters and hydrolases are the key factors that need to be induced, as they are responsible for transport and the first steps in the catabolism of the alternative sugars. However, Perez-Samper and colleagues used the molecular toolbox of *Saccharomyces cerevisiae* to identify the genes and processes that determine the length of the lag phase (Perez-Samper et al. 2018). The results show that while expression of transporters and hydrolases, such as the *MAL* or *GAL* genes which allow uptake and metabolism of maltose or galactose, respectively, is necessary for cells to escape the lag phase, the induction of these genes does not seem to be the rate-limiting step. Instead, Bar-Seq and transcriptome analyses reveal that efficient escape from the lag phase requires the activation of genes involved in respiratory metabolism. When *S. cerevisiae* is grown in glucose, respiratory metabolism is suppressed in favor of fermentation (De Deken 1966; Hagman et al. 2014). This so-called Crabtree effect is very similar to the Warburg effect in mammalian cells and likely allows cells to reach a maximal energy production rate. This, however, at the cost of efficiency, as fermentation of glucose yields more ATP molecules per minute, but fewer ATP molecules per molecule of glucose (Alexander and Jeffries 1990; Van Hoek et al. 1998; Vander Heiden et al. 2009; Diaz-Ruiz et al. 2011; Pfeiffer and Morley 2014). The results of Perez-Samper et al. show that when cells are shifted from glucose to an alternative carbon source such as galactose, induction of respiratory metabolism always precedes escape from the lag phase. Blocking respiration still allows this escape, but only after a much longer lag phase. Conversely, overexpressing *HAP4*, a master regulator of respiration in *S. cerevisiae*, shortens the lag phase significantly (Perez-Samper et al. 2018).

The duration of the lag phase also depends on the genetic makeup of a particular individual. The study by Perez-Samper et al. shows that different yeast strains exhibit large variation in their lag durations. Interestingly, these differences are correlated with the cellular concentration of proteins involved in respiration. Yeast strains that on average contain higher concentrations of respiration-associated proteins, especially proteins linked to the electron transport chain complexes, show shorter lag phases, again hinting at a central role of respiration as a key determinant of lag duration (Fig. 1b).

## History-dependent behavior: how the past influences the present

**Fig. 2:**
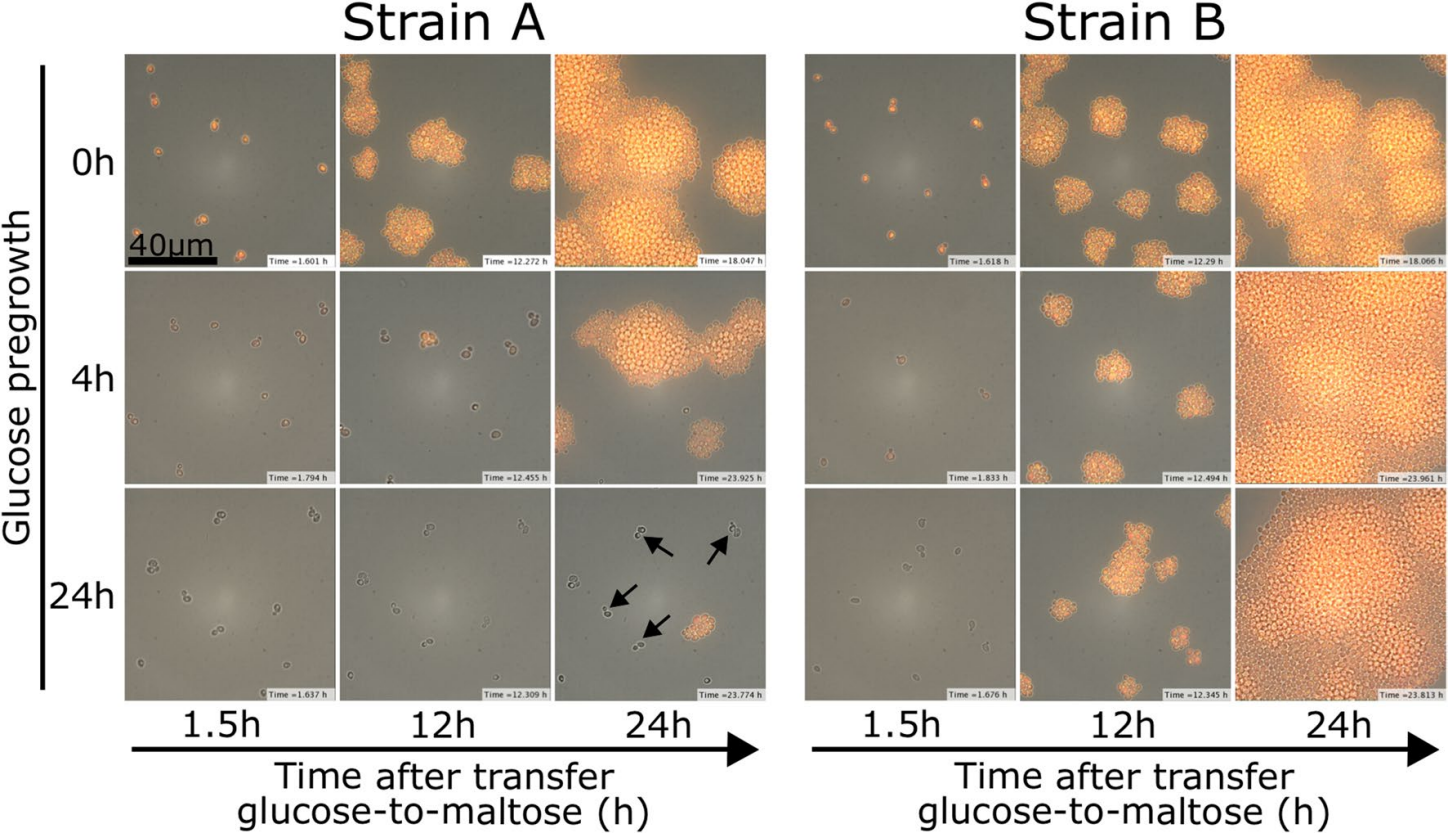
Time-lapse microscopy showing natural variation, history-dependent behavior and heterogeneity for two strains A and B after a sudden glucose-to-maltose shift. The induction of Mal12-yECitrine fluorescence can be used as a proxy for lag time. The rows show three different pregrowth conditions (0 h – 4 h – 24 h glucose pregrowth), the columns different time points during the microscopy experiment (1.5 h – 12 h – 24 h). The long-lag strain A induces Mal12, the maltose-cleaving enzyme, later than strain B, a short-lag strain. Longer glucose pregrowth leads to longer lag phases for both strains. Within the population, some cells induce Mal12 earlier than others. For strain A, some cells do not induce Mal12 even after 24 h (black arrows) and thus do not start to grow on maltose

In a second study, Cerulus and coworkers used live-cell microscopy to obtain a more detailed view on the lag behavior of *S. cerevisiae* cells (Fig. 2), measuring the lag duration of hundreds of individual cells in transitions from glucose to less preferred carbon sources such as maltose and galactose. They indeed confirm the previous finding of Perez-Samper et al. that the duration of the lag phase depends on the genetic background of a given yeast strain. Furthermore, they show that the lag duration is determined by the past environments that the cells encountered. Specifically, the results show that cells that have been growing in glucose for at least 12 h show much longer lag phases compared to cells that only grew on glucose for a few hours (Online Resource 1) (Cerulus et al. 2018). Interestingly, even daughter cells that have never directly seen the initial environment show this history-dependent behavior.

Previous studies hinted that such history-dependent behavior, sometimes referred to as “memory” or “hysteresis”, may be due to changes in the speed with which genes can be re-activated. For example, it has been proposed that the *S. cerevisiae GAL* genes can be re-activated more rapidly when cells were exposed to galactose in the previous few hours (Kundu and Peterson 2010; New et al. 2014; Stockwell et al. 2015). The key idea is that activation of these genes causes certain epigenetic changes, such as loosening of the chromatin structure around the promoters and/or posttranscriptional modifications to local nucleosomes, that are only slowly restored when the cells are transferred to conditions in which these genes are not induced (Turner 2002; Zacharioudakis et al. 2007; Tan-Wong et al. 2009; Brickner 2010; Kundu and Peterson 2010; Stockwell et al. 2015; D’Urso et al. 2016). Therefore, when cells are shifted from galactose to glucose and back to galactose, the *GAL* genes may, in theory, be induced more quickly if galactose is re-introduced before the chromatin structure around the *GAL* genes returns to its non-active state. Though plausible, there is little strong experimental evidence supporting this proposed mechanism. A second, alternative explanation for history-dependent behavior is transgenerational persistence of proteins. This proposed mechanism assumes that when cells are shifted from galactose to glucose, key Gal proteins are not actively broken down and, therefore, linger, only slowly disappearing because of natural degradation and dilution during cell division (Zacharioudakis et al. 2007; Stockwell et al. 2015). Interestingly, however, in the conditions used by Cerulus et al., the authors show that neither *GAL* or *MAL* gene induction nor the inheritance of Gal or Mal proteins drives the history-dependent behavior. When cells are shifted from galactose to glucose and back to galactose, the level of cellular Gal proteins at the time of the return to galactose indeed correlates nicely with observed lag times. This makes it tempting to speculate that Gal protein inheritance is indeed at the basis of the history-dependent behavior. However, when cells are switched from maltose to glucose to galactose, the lag phases upon the switch to galactose are similar to the galactose–glucose–galactose lag phases, even though the first growth phase in maltose does not induce *GAL* gene expression, and cells do not carry detectable levels of Gal proteins (Cerulus et al. 2018). Instead, the authors, using several genome-wide screens, hint at a central role for the activation of respiration as a crucial factor to escape the lag phase. Similar to what Perez-Samper et al. report on the population level, single-cell analyses confirm that genes involved in respiratory metabolism are activated prior to escape from the lag phase, and even prior to activation of the *GAL* or *MAL* genes. Blocking respiration lengthens the lag phase, while over-activating respiration by overexpressing *HAP4* results in shorter lag phases. Hence, it seems that, instead of transcriptional memory in *GAL* or *MAL* gene expression or inheritance of Mal or Gal proteins, the expression or inheritance of proteins and complexes involved in respiration may be the key factor underlying history-dependent behavior in the lag phase.

It seems plausible that when cells are shifted to glucose, respiration is only slowly repressed, with full repression taking about 12 h, equivalent to about 6–8 cell divisions. If cells are shifted back to an alternative carbon source before respiration is completely repressed, they are able to more rapidly adjust and resume growth (Fig. 1b). However, thus far, the exact genes or molecules involved have not yet been completely identified. Recently, a molecular mechanism for a rapid, adaptive response through regulated protein aggregation of respiratory activators was proposed, which could also influence history-dependent behavior (Simpson-Lavy and Kupiec 2018). Moreover, it is still unclear why cells need to activate respiration because, in principle, sugars such as maltose and galactose can also be fermented. However, during a switch in carbon sources, cells experience a severe drop in intracellular ATP levels, and this drop is much more severe in cells that cannot respire (Perez-Samper et al. 2018). Hence, a threshold level of respiration may allow cells to efficiently produce energy from the few molecules of sugar they can import and/or from reserve carbohydrates that are stored intracellularly, providing them with the resources needed to reprogram their metabolism and resume growth.

## The lag phase differs between individual cells

Another aspect of the lag phase revealed through single-cell analyses is that despite being genetically identical, individual cells in a population often show very different behaviors (Fig. 1b). While some cells are able to escape the lag phase a couple of hours after the shift, other cells take more than 20 h (Cerulus et al. 2018). Even more surprisingly, a significant fraction of the cells seems to never escape the lag phase at all. In some conditions and for some genetic backgrounds, the fraction of cells that fails to escape the lag phase is more than 90%. This is quite puzzling, as one would intuitively expect that cells would have evolved to respond as quickly as possible to environmental changes. After all, cells that adapt more rapidly can escape the lag phase and resume growth more quickly, thereby outcompeting cells that take longer to escape. Moreover, it seems logical to assume that genetically identical cells in a uniform environment would respond similarly to environmental triggers. Why, then, are some cells in an isogenic population, subjected to exactly the same environment, able to switch rapidly whereas some others even completely fail to escape the lag phase?

One simple explanation would be that the lag duration depends on the cell cycle stage a particular cell is in when the carbon source shift happens. Another possibility would be that older mother cells, that have been growing in glucose much longer than newly born daughter cells, show slower transitions. However, no dependency on cell cycle or replicative age was found (Cerulus et al. 2018). What, then, could explain the differences between individual cells? One hypothesis is that during growth on glucose, some cells show stronger repression of respiration than others. Cells that are partly respiring may show shorter lag phases, possibly at the cost of fitness during glucose growth. Indeed, it has been shown that strong catabolite repression leads to optimal fitness in stable glucose environments, whereas a more relaxed repression may be more favorable in variable and unpredictable environments (Fig. 1b) (New et al. 2014; Wang et al. 2015). So, depending on the specific conditions, it might be beneficial for cells to not switch too quickly when the environment changes.

Interestingly, various single-cell analyses have demonstrated the existence of seemingly stochastic differences between genetically identical cells. These differences, often referred to as biological noise, can result from various sources, not in the least stochastic processes related to molecular interactions, gene expression, and protein stability and inheritance (Newman et al. 2006; Maheshri and O’Shea 2007; New et al. 2014). Moreover, theoretical work suggests that a quick and uniform response is perhaps not always the best strategy, and that noise may in some cases be beneficial. For example, a seminal study by Kussell and Leibler predicts that in some cases it may be more favorable if cells in a population do not uniformly adapt to an environmental change, but instead employ stochastic switching between different states, with each state fitting a particular environment (Kussell and Leibler 2005). This implies that in any given population and any given environment, a fraction of the cells is not optimally adapted. However, the flipside of this strategy is that a population always contains a certain fraction of cells that can resume growth as soon as a new environment comes along. In addition, mathematical models suggest that the fitness effect of a fraction of slow-growing cells in a population is much smaller than one would intuitively predict (Cerulus et al. 2016). The proposed stochastic switching would be most favorable when the environmental changes are also stochastic and infrequent since the cost to maintain a sensing and signaling system becomes larger if having the sensor only becomes useful occasionally. Of course, much also depends on the switching rates of the stochastic system; these are presumed to be evolutionarily tuned to the rate at which the environmental changes take place. Similarly, theoretical work also predicts that a form of epigenetic memory or hysteresis may also help to further tune the adaptive response of cells (Kussell and Leibler 2005; Friedman et al. 2014). In this scenario, prolonged exposure to a given environment is interpreted as a sign that this environment has a high chance of returning, making it advantageous to not have all individuals quickly shift to a new state when a (temporary) change occurs.

However, it is unclear whether the lag phase variation between individual cells is indeed adaptive rather than merely a consequence of biological noise. In that respect, it would be interesting to compete with otherwise isogenic variants that show different lag behaviors, ranging from uniformly short lags to highly heterogeneous long lags, in various environments. Would it indeed be true that strains showing more history-dependent behavior and heterogeneous lag phases show increased fitness in highly variable and unpredictable environments? To investigate this, one would first need to identify the natural alleles that explain variation in lag behavior, for example, by performing a Quantitative Trait Locus (QTL) analysis, starting from a few fast- and slow-switching strains. Then by introducing only those mutations that cause the difference in lag behavior, one would have a perfect set of variants to study the implications of having short or long lag times.

Together, the new results show that, while activation of specific genes involved in the uptake and metabolism of alternative sugars, such as the *GAL* and *MAL* genes, is necessary to resume growth on alternative carbon sources after glucose runs out, the cells first need to make a bigger change in their metabolism, namely re-routing carbon flux from fermentation to respiration (Perez-Samper et al. 2018; Cerulus et al. 2018). This major re-routing is, in at least some cases, the rate-limiting step, and the longer cells have grown on glucose, and the more their metabolism is adapted to it, the more difficult and slow a switch to a new carbon source becomes, even for cells that were born in the last few hours or minutes before the switch from glucose to maltose or galactose. Interestingly, the heterogeneity in the lag phase between different yeast strains and between different cells in the same population suggests that the lag phase might be evolutionary optimized through various mechanisms, including genetic mechanisms that determine the strength of repression of respiration in glucose (New et al. 2014), as well as the level of stochasticity in the response (Cerulus et al. 2016, 2018). In light of these new results from Perez-Samper et al. and Cerulus et al. (2018), it might also be interesting to investigate whether this re-routing from fermentation to respiration can be linked to mechanisms behind other important biological phenomena such as stress tolerance, longevity and quiescence (Miles and Breeden 2017; Soontorngun 2017; Zhang and Cao 2017; Pascual-Ahuir et al. 2018).

## Electronic supplementary material

Below is the link to the electronic supplementary material.

Online Resource 1 Video of time-lapse microscopy showing natural variation, history-dependent behavior and heterogeneity for two strains A and B after a sudden glucose-to-maltose shift. The induction of Mal12-yECitrine fluorescence can be used as a proxy for lag time. The top row shows the lag and subsequent growth on maltose for the long-lag strain A for five different pregrowth conditions (0 h – 2 h – 4 h – 6 h – 24 h glucose pregrowth). The bottom row shows the lag and subsequent growth on maltose for the short-lag strain B for the same five pregrowth conditions. Longer glucose pregrowth conditions lead to longer lag phases for both strains, with more extreme effects for strain A. Within the population, heterogeneity in lag duration can be seen, again with more extreme effects for strain A, where after 24 h some cells have not induced Mal12 and thus have not started to grow on maltose. (AVI 610874 KB)

## References

[CR1] Alexander MA, Jeffries TW (1990). Respiratory efficiency and metabolite partitioning as regulatory phenomena in yeasts. Enzyme Microb Technol.

[CR2] Brickner JH (2010). Transcriptional memory: staying in the loop. Curr Biol.

[CR3] Cerulus B, New AM, Pougach K, Verstrepen KJ (2016). Noise and epigenetic inheritance of single-cell division times influence population fitness. Curr Biol.

[CR4] Cerulus B, Jariani A, Perez-Samper G (2018). Transition between fermentation and respiration determines history-dependent behavior in fluctuating carbon sources. Elife.

[CR5] D’Urso A, Takahashi Y, Xiong B (2016). Set1/COMPASS and mediator are repurposed to promote epigenetic transcriptional memory. Elife.

[CR6] De Deken RH (1966). The Crabtree effect: a regulatory system in yeast. J Gen Microbiol.

[CR7] Diaz-Ruiz R, Rigoulet M, Devin A (2011). The warburg and crabtree effects: on the origin of cancer cell energy metabolism and of yeast glucose repression. Biochim Biophys Acta Bioenerg.

[CR8] Friedman G, McCarthy S, Rachinskii D (2014). Hysteresis can grant fitness in stochastically varying environment. PLoS One.

[CR9] Hagman A, Säll T, Piškur J (2014). Analysis of the yeast short-term Crabtree effect and its origin. FEBS J.

[CR10] Jacob F, Monod J (1961). Genetic regulatory mechanisms in the synthesis of proteins. J Mol Biol.

[CR11] Kundu S, Peterson CL (2010). Dominant role for signal transduction in the transcriptional memory of yeast GAL genes. Mol Cell Biol.

[CR12] Kussell E, Leibler S (2005). Phenotypic diversity, population growth, and information in fluctuating environments. Science.

[CR13] Maheshri N, O’Shea EK (2007). Living with noisy genes: how cells function reliably with inherent variability in gene expression. Annu Rev Biophys Biomol Struct.

[CR14] Miles S, Breeden L (2017). A common strategy for initiating the transition from proliferation to quiescence. Curr Genet.

[CR15] New AM, Cerulus B, Govers SK (2014). Different levels of catabolite repression optimize growth in stable and variable environments. PLoS Biol.

[CR16] Newman JRS, Ghaemmaghami S, Ihmels J (2006). Single-cell proteomic analysis of S. cerevisiae reveals the architecture of biological noise. Nature.

[CR17] Pascual-Ahuir A, Manzanares-Estreder S, Timón-Gómez A, Proft M (2018). Ask yeast how to burn your fats: lessons learned from the metabolic adaptation to salt stress. Curr Genet.

[CR18] Perez-Samper G, Cerulus B, Jariani A (2018). The Crabtree effect shapes the *Saccharomyces cerevisiae* lag phase during the switch between different carbon sources. MBio.

[CR19] Pfeiffer T, Morley A (2014). An evolutionary perspective on the Crabtree effect. Front Mol Biosci.

[CR20] Simpson-Lavy K, Kupiec M (2018). A reversible liquid drop aggregation controls glucose response in yeast. Curr Genet.

[CR21] Soontorngun N (2017). Reprogramming of nonfermentative metabolism by stress-responsive transcription factors in the yeast *Saccharomyces cerevisiae*. Curr Genet.

[CR22] Stockwell SR, Landry CR, Rifkin SA (2015). The yeast galactose network as a quantitative model for cellular memory. Mol Biosyst.

[CR23] Tan-Wong SM, Wijayatilake HD, Proudfoot NJ (2009). Gene loops function to maintain transcriptional memory through interaction with the nuclear pore complex. Genes Dev.

[CR24] Turner BM (2002). Cellular memory and the histone code. Cell.

[CR25] Van Hoek P, Van Dijken JP, Pronk JT (1998). Effect of specific growth rate on fermentative capacity of baker’s yeast. Appl Environ Microbiol.

[CR26] Vander Heiden MG, Cantley LC, Thompson CB (2009). Understanding the Warburg effect: the metabolic requirements of cell proliferation. Science.

[CR27] Wang J, Atolia E, Hua B (2015). Natural variation in preparation for nutrient depletion reveals a cost-benefit tradeoff. PLoS Biol.

[CR28] Zacharioudakis I, Gligoris T, Tzamarias D (2007). A yeast catabolic enzyme controls transcriptional memory. Curr Biol.

[CR29] Zhang N, Cao L (2017). Starvation signals in yeast are integrated to coordinate metabolic reprogramming and stress response to ensure longevity. Curr Genet.

